# Urine Monocyte Chemoattractant Protein-1(UMCP-1) as a Biomarker of Renal Involvement in Systemic Lupus Erythematosus

**Published:** 2012

**Authors:** Zahra Mirfeizi, Mahmoud Mahmoudi, Masih Naghibi, Mohammmadreza Hatef, Farzaneh Sharifipour, Mohammadhassan Jokar, Abbasali Zeraati, Zhaleh Shariati Sarabi, AmirAbbas Azarian

**Affiliations:** 1 *Rheumatic Diseases Research Centre (RDRC), Mashhad University of Medical Sciences, Mashhad, Iran*; 2 *Immunology Research Centre (IRC), Mashhad University of Medical Sciences, Mashhad, Iran*; 3 *Imam Reza Hospital, Mashhad University of Medical Sciences, Mashhad, Iran*; 4 *Rheumatic Diseases Research Centre (RDRC), Mashhad University of Medical Sciences, Mashhad, Iran*; 5 *Imam Reza Hospital, Mashhad University of Medical Sciences, Mashhad, Iran*; 6 *Vice Chancellor for Research Office, Mashhad University of Medical Sciences, Mashhad, Iran*

**Keywords:** Lupus nephritis, Monocyte chemoattractant protein 1, Systemic lupus erythematosus, Urine

## Abstract

**Objective(s):**

Lupus nephritis (LN) is frequently associated with a poor long-term prognosis. Renal biopsy is the diagnostic method of choice in this condition. Urine biomarkers have been mentioned in the diagnosis of LN. The study^,^s purpose was to evaluate the performance of urinary monocyte chemoattractant protein 1(UMCP-1) as a biomarker of renal involvement in systemic lupus erythematosus.

**Materials and Methods:**

Forty-one recently diagnosed systemic lupus erythematosus patients (8 male and 33 female) without renal involvement (group 1) and twenty six patients (8 male and 18 female) with LN (group 2), proven by biopsy, were recruited to this study. UMCP-1 sensitivity and specificity for identifying biopsy-proven nephritis were calculated, and a receiver operating characteristic (ROC) curve was constructed to quantify how definitely UMCP-1 distinguishes between patients with and without LN.

**Results:**

The mean value of UMCP-1 levels were 733.07 pg/ml ± 1282.54 and 144.16 pg/ml ± 137.90 in patients with and without LN respectively. The UMCP-1 level was significantly higher in group 2 than group 1. There was no significant correlation between UMCP-1 and 24-hour urine protein (r = 0.031, *P*= 0.874). The area under the ROC curve was 0.727 with a CI 95% of 0.597 to 0.857 (*P*=0.002). Using a cut-off value of 82 pg/ml,UMCP-1 had a sensitivity of 88.5% and a specificity of 46.3% for identifying LN.

**Conclusion:**

UMCP-1 can serve as a biomarker of LN although further longitudinal studies of these biomarkers are required in LN.

## Introduction

Systemic lupus erythematosus (SLE) is a common autoimmune disease that can affect every organ in the body. Renal involvement becomes clinically apparent in approximately 75 percent of patients; however, most of the remaining patients have subclinical disease that can be demonstrated if renal biopsy were performed. Renal involvement usually develops in the first few years of illness, and should be detected early by periodic urinalyses and estimation of the glomerular filtration rate (1). 

There is a spectrum of renal injury that can be assessed, in part on clinical grounds, and more definitely by biopsy. Initial categories of LN were based on classification by the World Health Organization (WHO) as assessed by histology and location of immune complexes. Recently, this classification has been revised by the International Society of Nephrology and Renal Pathology Society (ISN/RPS) (2). This system divides the glomerular disorders into six different patterns or classes. A renal biopsy is necessary to make a definite diagnosis of histologic patterns of renal involvement. Although renal biopsy remains the gold standard for diagnosis of LN, it is an invasive method and has potential complications such as bleeding and even death (3). New laboratory tests are needed to identify renal involvement without renal biopsy.

Blood and urinary biomarkers can be used for initial detection of renal involvement in SLE. Urinary monocyte chemoattractant protein-1(UMCP-1) has been proposed as a potential predictor of renal involvement in lupus patients (3). MCP-1 is produced by renal mesangial, endothelial, tubuloepithelial and smooth muscle cells (4). MCP-1 attracts and activates monocyte and T cells in acute inflammatory conditions, and is also an important mediator in chronic inflammation (5). There are some evidences in both human and animal studies regarding the role of MCP-1 for renal injury in lupus patients (6, 7). For the current study, we hypothesized that UMCP-1 may also represent a novel biomarker for the identification of LN. The aim of the present study was to evaluate the role of UMCP-1 as a biomarker of renal involvement and its predictive values of histological patterns of renal involvement.

## Materials and Methods

 Sixty seven recently diagnosed Iranian SLE patients were enrolled in this study . None of patients had received immunosuppressive agents. All patients had SLE according to the American College of Rheumatology criteria for diagnosis of SLE (8). They were recruited consecutively as new patients with SLE who attended the Imam Reza Hospital, Mashhad, Iran. They were divided into two groups.

Group 1:

There were 41 patients (8 male and 33 female) with SLE without clinically apparent renal involvement. 

Group 2:

This group included 26 patients (8 male and 18 female) with SLE and recently proven renal involvement by biopsy. All renal biopsies were performed as a routine diagnostic procedure. Renal biopsy specimens were analyzed and scored according to the 2003 ISN/RPS classification of LN . 

 All patients underwent routine laboratory assessments. Blood samples were obtained for determination of the complete blood cell count, serum creatinine, serum C3, C4, ANA, anti ds-DNA. All patients, regardless of the severity of the disease, were asked to provide a 24-hr urine collection for determination of proteinuria. The subjects were advised to pass urine at 8:00 am and collect all the urine subsequently till 8:00 am the next morning (24 hr period). The adequacy of urine collection was determined by creatinine excretion.

***Urinary MCP-1 measurement***

Urine spot samples were stored in -70ºC until performing the experiments. Samples were brought to room temperature for testing. UMCP-1 concentrations were determined using human MCP-1 ELISA kit (Bender Medsystems TM, Austria) according to the manufacturer’s instruction. Each sample was tested in duplicates. The intra-assay and inter-assay coefficient of variation were 4.7 and 6.2%, respectively.

***Statistical Analysis***

Statistical analysis was performed with SPSS for Windows software version 11.5 (SPSS Inc., Chicago, IL, USA). Mean and standard deviations were used to express quantitative data. Comparison between means of two groups was done by using Student's *t*-test. ANOVA test was performed for analysis of variances. Correlation between MCP-1 and 24 hr urine protein secretion were analyzed using Pearson correlation coefficient. UMCP-1 sensitivity and specificity for identifying biopsy-proven nephritis were calculated, and a receiver operating characteristic (ROC) curve was constructed. *P* values of <0.05 were considered statistically significant.

***Ethics***

Ethical approval for this study was obtained from the Ethical Committee of Mashhad University of Medical Sciences- Vice- Chancellor for Research (code:86109). Written informed consent was obtained from all subjects. 

## Results

There were 41 patients (8 male and 33 female) in group 1 and 26 patients (8 male and 18 female) in group 2. The mean age was 28 ± 5.5 years in group 1 and 27 ± 6 years in group 2. ANA was positive in 98% and Anti ds-DNA dsDNA in 91% of all patients (Table 1). There was no significant difference regarding age (*P*= 0.52) and sex (*P*= 0.38) between two groups. The mean value of UMCP-1 levels in group 1 and group 2 were 144.16 ± 137.90 and 733.07 ± 1282.54 pg/ml respectively. In general UMCP-1 level was significantly higher in group 2 (with lupus nephritis) than in group 1 (without lupus nephritis) (*P* = 0.028). The UMCP-1 were significantly higher in females with LN compared to those without LN (*P* P= 0.004). However, it was not significant in males (*P* = 0.28) (Table 2). 

**Figure 1:**
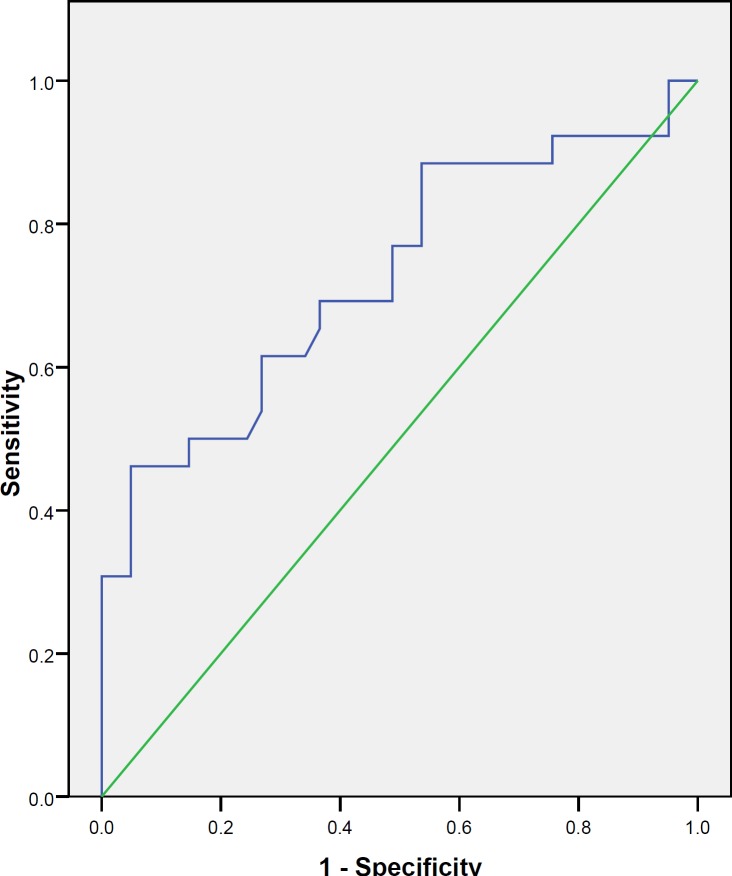
Roc curve of UMCP-1 represents AUC=0.727 for detecting of lupus nephritis

 There was no correlation between UMCP-1 and 24-hr urine protein (r_p_ =0.03, *P* =0.874). A nonparametric ROC curve, constructed to quantify how definitely UMCP-1 distinguishes between SLE patients with and without nephritis, showed an AUC of 0.727 with a CI 95% of 0.597 to 0.857 (*P *= 0.002; Figure 1). According to a cut-off point of 82 pg/ml, UMCP-1 showed a sensitivity of 88.5% and a specificity of 46.3% for identifying LN.

**Table 1:** Patient's demographics and some laboratory parameters of studied patients

Characteristics	Group 1	Group 2	*P*-value (Statistical test)
Number of participants	41	26	
Gender (%)			
Male Female	8 33	8 18	0.38(chi square)
Age, y	28±5.5	27±6	0.52 (T test)
Positive anti ds-DNA N (%)	36 (87)	25 (96)	0.23(chi square)
Positive ANA N (%)	41 (100)	25 (96)	0.21(chi square)

**Table 2:** UMCP-1 level in studied groups considering gender

Groups	SLE without renal involvement Mean (SD)	SLE & renal involvement Mean (SD)	Mann-Whitney test *P-* value
Female	147.98 pg/ml (146.39)	853.41 pg/ml (1490.03)	0.004*
Male	128.4 pg/ml (101.70)	462.32 pg/ml (601.51)	0.279

* Significant

## Discussion

Early detection of renal involvement during the course of SLE is important for improving the outcome (1). Kidney biopsy is usually regarded as the ‘gold standard’ for diagnosis and histological classification of LN. Nonetheless, there are important problems of kidney biopsy (3). It has thus become clear that there is a real need for investigating surrogate markers to predict the degree of renal involvement. There are some evidences that UMCP-1 is a biomarker for LN (6, 7). 

 In the present study, we investigated the UMCP-1 in SLE patients with and without nephritis. The UMCP-1 level was significantly higher in patients with lupus nephritis than patients without lupus nephritis. However, there was not a significant correlation between UMCP-1 and proteinuria. 

This finding was in agreement with some other studies. In the study of Wada *et al* (9), it was shown that UMCP-1 in patients with LN was significantly higher than patient without LN. In another study, Tucci *et al* (10) examined the role of a functional MCP-1 polymorphism in SLE and LN. They showed that, U MCP-1 values were significantly higher in patients with LN. In the study by Rowin *et al* (11), they found that in renal flares, mean UMCP-1 values were significantly higher than UMCP-1 in non renal flares.These values were also higher in comparison with healthy volunteers and other causes of renal involvements as a control group. In the study conducted by Liu *et al* (12), the serum MCP-1 levels were measured in 112 patients with SLE, 30 patients with rheumatoid arthritis, 11 non-SLE patients with renal impairment, and 40 healthy volunteers. The expression of MCP-1 was significantly higher in active LN groups than in all other groups, and there was a close correlation between MCP-1 expression and the overall SLE disease activity index score and the SLE disease activity index renal score. After activation, mesangial cells can express MCP-1 by growth factors. In addition to mesangial cells, endothelial cells and infiltrating mononuclear cells also express MCP-1. Tubular epithelial cells seem to be the major source of MCP-1 in urine. The increased production of MCP-1 by tubular epithelial cells is due to the stimulation by cytokines and exposure to urinary proteins. We illustrated that UMCP-1 levels were significantly higher in females with LN compared to those without LN, while there was no statistical difference between the mean levels of UMCP-1in these groups in males. The main reason for this issue was probably the fact that, in comparison with females in our series, the number of men was not adequate to make a reliable calculation. Although previous results firmly support the notion that UMCP-1 is very sensitive and specific for identifying LN, however, we found that UMCP-1 had high sensitivity and low specificity for predicting LN in the research’s adult patients with lupus. There are some problems in the clinical utility of UMCP-1. It may stem from its nonspecific nature; for example urinary MCP-1 levels also rise with various other types of renal injuries, including ischemic and toxic injury. Proteinuria stimulates renal tubular epithelial cells to produce cytokines such as MCP-1 that can contribute to chronic kidney disease with increasing proteinuria in adult LN. However there wasn’t any significant correlation between UMCP-1 and proteinuria in our study.

 There are some evidences that locally produced MCP-1 plays a role in the initiation and progression of tubulointerstitial damage. However, MCP-1 seems to be particularly involved in the progression of glomerular lesions. Notably, raised urinary levels of MCP-1 have been reported in humans or animals alongside with kidney disease, and this correlates with the degree of urinary protein excretion (13). Nevertheless, in contrast to some studies, we did not find any correlation between UMCP-1 and 24-hr urinary protein excretion. In a study by Tucci *et al * (10) they showed that UMCP-1 and 24-hr urinary protein excretion have positive correlation. Marks *et al* studied glomerular expression of MCP-1 in pediatric lupus nephritis (14). They showed a correlation between increased glomerular expression of MCP-1 and albuminuria. In another study, Kim *et al* observed a positive correlation between urinary excretion of MCP-1 and proteinuria (16). In correspondence with the present study, Chunsun *et al* could not find any correlation between UMCP-1 and extent of urinary protein excretion in patients with LN (15).

 Certain limitations encountered in the process which need to be addressed in the future; first, the small sample size which causes the unreliability of the subgroup comparison of UMCP-1 among different histological classes of lupus nephritis. In this regard, future investigations on serial measurement of UMCP-1 together with its correlation with disease activity and treatment response will be needed. Comparative studies between UMCP-1 and current disease activity markers such as anti ds-DNA and complement are also encouraged in future investigations.

## Conclusions

These preliminary results suggest that UMCP-1 might be used as a biomarker for assessing disease activity and risk stratification in LN. Future studies are needed to define the role of this marker. We showed here that UMCP-1 can serve as a biomarker of LN although further longitudinal studies of these biomarkers are required in LN.
